# Keratinocyte-to-macrophage communication exacerbate psoriasiform dermatitis via LRG1-enriched extracellular vesicles

**DOI:** 10.7150/thno.89180

**Published:** 2024-01-01

**Authors:** Wenjuan Jiang, Tingting Zhang, Yueqi Qiu, Qianmei Liu, Xiaoyun Chen, Qiaolin Wang, Xiaoli Min, Lianlian Ouyang, Sujie Jia, Qianjin Lu, Yuan He, Ming Zhao

**Affiliations:** 1 Institute of Dermatology, Chinese Academy of Medical Sciences and Peking Union Medical College, Nanjing, China.; 2 Key Laboratory of Basic and Translational Research on Immune-Mediated Skin Diseases, Institute of Dermatology, Chinese Academy of Medical Sciences, Nanjing, China.; 3 State Key Laboratory of Natural Medicines, School of Basic Medicine and Clinical Pharmacy, China Pharmaceutical University, Nanjing, China.; 4 Department of Dermatology, The Second Xiangya Hospital of Central South University, Changsha, China.

**Keywords:** Psoriasis, Extracellular vesicle, Leucine-rich α-2-glycoprotein 1, Macrophage, Keratinocyte.

## Abstract

**Rationale:** Macrophage-associated inflammation and keratinocytes excessive proliferation and inflammatory cytokines secretion induced by stimulation play an important role in the progression of psoriasiform dermatitis. However, how these two types of cells communicate remains obscure.

**Methods:** We induced a mouse model with experimental psoriasiform dermatitis by Imiquimod (IMQ). To investigate whether damaged keratinocytes promote macrophage polarization and accelerate skin lesions by releasing extracellular vesicle (EV), purified EV were isolated from the primary epidermis of 5-day IMQ-induced psoriasiform dermatitis model mice, and then fluorescence-labeled the EV with PKH67. The EV was injected into the skin of mice treated with IMQ or vehicle 2 days *in situ*. In addition, we established a co-culture system of the human monocytic cell line (THP-1) and HaCaT, and THP-1/HaCaT conditioned media culture model *in vitro* respectively. Subsequently, we evaluated the effect of Leucine-rich α-2-glycoprotein 1 (LRG1)-enriched EV on macrophage activation.

**Results:** We demonstrated macrophages can significantly promote keratinocyte inflammation and macrophage polarization may be mediated by intercellular communication with keratinocytes. Interestingly, IMQ-induced 5-day, keratinocyte-derived EV recruited macrophage and enhanced the progression of skin lesions. Similar to results *in vivo*, EV released from M5-treated HaCaT significantly promotes Interleukin 1β (IL-1β) and Tumor necrosis factor α (TNF-α) expression of THP-1 cells. Importantly, we found that LRG1-enriched EV regulates macrophages via TGF beta Receptor 1 (TGFβR1) dependent process.

**Conclusion:** Our findings indicated a novel mechanism for promoting psoriasiform dermatitis, which could be a potential therapeutic target.

## Introduction

Psoriasis is an inflammatory skin disease that is associated with many other medical conditions and affects over 60 million adults and children worldwide 1, 2. Although the pathogenesis of psoriasis remains controversial, it is generally accepted that the complex communication between activated keratinocytes and infiltrated immune cells leads to disease development 3. Reducing immune cell infiltration is the key to effective treatment of psoriasis.

Recent studies have found that removing macrophages could improve the severity of psoriasis, indicating that the collection and activation of macrophages play an important role in the onset of psoriasis 4. Selective removal of macrophages not only improved clinical symptoms but also reduced Th1 cytokine levels 5. It was also found that psoriasis could be reduced by inhibiting the inflammation of macrophages 6. Just as the application of biological agents that inhibit Tumor necrosis factor α (TNF-α), it also significantly reduced the proportion of macrophages in skin lesions in patients with psoriasis and the proportion of M1 macrophages 7, 8. However, at present, the regulatory mechanism of the activation of macrophages in psoriasis lesions has not been clarified, and the correlation between regulating macrophages and the severity of skin lesions needs to be further explored.

Beyond classical signaling through cell-cell contact and soluble factors, such as cytokines, inflammatory mediators, metabolites, and hormones, such intercellular communication also occurs through cellular release of extracellular vesicle (EV). This mode of communication has the potential to deliver a particularly diverse array of messages to EV-accepting cells at a level beyond that of soluble factor signaling, since EV may carry a number of bioactive molecules, surface receptors, and genetic information 9, 10. In the past, many studies have reported that the communication between dendritic cells, T cells and keratinocytes in the skin lesions of psoriasis promoted the skin lesions of psoriasis 11, 12. Studies have reported that EV from keratinocytes have also been found to regulate neutrophils to promote psoriasis skin inflammation 13. It suggests that the EV from keratinocytes can participate in the development of psoriasis, but its mechanism and how to regulate other immune cells are still unknown.

In the current study, we provide evidence suggesting that keratinocyte-derived EV induced expression and release of proinflammatory cytokines, such as Interleukin 1β (IL-1β) and TNF-α, in macrophages. Importantly, we found that Leucine-rich α-2-glycoprotein 1 (LRG1)-enriched EV polarizes macrophages via TGF beta Receptor 1 (TGFβR1) dependent process. Taken together, keratinocytes-to-macrophages communication via EV transfer promotes psoriasiform dermatitis.

## Materials and Methods

### Experimental reagents

β-actin, TGFβR1 and goat anti-rabbit or anti-mouse immunoglobulin G (IgG) horseradish peroxidase (HRP) secondary antibodies were purchased from Bioss (Beijing, China). Antibodies against Tumor susceptibility gene 101 (TSG101), CD63, Adenosine diphosphate ribosylation factor 6 (ARF6), Calnexin, and LRG1 were obtained from Abcam (Cambridge, UK). Cytochalasin D was obtained from APExBIO (USA). PKH67 was obtained from Sigma (St. Louis, MO). A83-01 and LY-364947 were purchased from MCE (USA).

### Mice

All mice experiments were performed with 6-8-week-old female BALB/c mice which were purchased from SPF (Nanjing, China) Biotechnology Co.Ltd. All animal experiments were performed following the Regulations of the Experimental Animal Administration issued by the State Committee of Science and Technology of China. All mouse experiments were approved by the Hospital for Skin Diseases, Institute of Dermatology, Chinese Academy of Medical Sciences & Peking Union Medical College in Nanjing, China. Mice were housed in specific pathogen-free conditions with a controlled environment at 24 ± 2℃, with a 12-hour light/dark cycle and 50 ± 10% humidity that food and water were free.

For the establishment of the psoriasis mouse model, mice were topically treated with 62.5 mg of commercially available 5% Imiquimod (IMQ) cream (Med-shine Pharma, Chengdu, China) on shaved 2.5 cm × 2.5 cm back skin daily for 0-7 consecutive days. Mice were sacrificed 1 day after the final treatment.

### Isolation and culture of mouse epidermis

Fresh Mouse skin from animal surgery was taken, washed 2-3 times with DPBS containing 1% antibiotics. The epidermis and dermis were separated by incubating in 2 mg/mL Dispase II protease (Sigma, USA) overnight at 4℃, and then epidermis was cultured in Keratinocyte-SFM (Gibco, USA) at 37℃, in 5% carbon dioxide, and 95% relative humidity. Keratinocyte-SFM is a complete serum-free medium when supplemented with human recombinant Epidermal Growth Factor (EGF) and Bovine Pituitary Extract (BPE) at the time of use. After 24 h of culture, cell supernatants were collected and centrifuged to remove cell debris, after which were collected for EV isolation.

### Cell lines

Human immortalized Keratinocyte cell line HaCaT were cultured in DMEM (Gibco, USA) supplemented with 10% FBS (HyClone, Logan, UT, USA) and 1% antibiotics at 37℃ in a humidified incubator under 5% carbon dioxide. The medium was refreshed every 2 days and cells were subcultured according to the cell fusion. Establishment of a psoriasis-like cell model induced by 10 ng/mL M5 (IL-17A; IL-22; TNF-a; Peprotech, Rocky Hill, NJ, USA) (IL-1α; Oncostatin M; Novoprotein, Shanghai, China) *in vitro*: HaCaT Cells at 40-60% confluence were cultured in FBS-free DMEM and stimulated with M5 for 24 h later. Cell culture supernatant was then collected for EV isolation, and EVs were collected for subsequent assays. The human monocytic cell line (THP-1), which can be differentiated into macrophage-like cells, were cultured in RPMI-1640 (HyClone, Logan, UT, USA) supplemented with 10% FBS (HyClone) at 37℃ in a humidified incubator under 5% carbon dioxide. For all experiments, THP-1 cells were induced to differentiate into macrophages using Phorbol 12-myristate 13-acetate (PMA, Sigma) at 10 ng/mL for 24 h before use in studies.

### Extracellular vesicle isolation

EV isolated from Psoriasis-like mouse primary keratinocyte cells and psoriasis-like HaCaT cells model were prepared by a commercially available ExoQuick-TC EV precipitation solution kit from System Biosciences (Palo Alto, CA) by collecting in EV-deficient complete medium. In brief, the collected cell supernatant was centrifuged at 3000 g for 15 min at 4°C to remove cells and cell debris, then precipitator was added overnight (at least 12 h), followed by centrifugation at 2000 g for 15 min to discard the supernatant. The obtained pellets were resuspended in PBS solution. EV solutions intended for cell treatment were sterile filtered through a 0.22 µm syringe filter. Resuspended EVs were either used for subsequent analysis or aliquoted and stored at -80℃.

### Electron microscopy

For electron microscopic visualization of EVs, the isolated EVs were fixed in 2% paraformaldehyde in 0.1 M phosphate buffer overnight at 4°C. The samples were then placed on a Formvar-carbon-coated grid and air dried for 20 min. After being rinsed with PBS, grids were transferred to 1% glutaraldehyde for 5 min and washed with distilled water. The grids were first contrasted with uranyl-oxalate solution and then contrasted and embedded in a mixture of 4% uranylacetate and 2% methylcellulose (1:9 ratio). The grids were air dried and visualized with a JEOL 1400 electron microscope (JEOL USA, Peabody, MA) at 80 kV. For immunogold staining, grids were blocked with 10% FBS for 20 min followed by overnight incubation at 4℃ with anti-LRG1 antibodies diluted 1:20 in blocking solution overnight. Next, grids were incubated with secondary antibody for 1 h. In negative control samples, primary antibody was omitted. Samples were then labeled with protein A-10-nm gold for 1 h. The grids were contrasted and embedded with a mixture of 4% uranyl acetate and 2% methylcellulose (1:9 ratio) and observed as described above.

### Nanoparticle tracking analysis

Particle size and concentration of isolated EVs were performed using Nanoparticle tracking analysis (NTA) on NanoSight NS300 instrument (NanoSight, Amesbury, UK). EV samples were diluted with PBS at a range of concentrations between 4×10^8^ and 8×10^8^ particles per milliliter in a total volume of 1 milliliter. Each sample was continuously run through a flow-cell top plate set up to 23.3℃ using a syringe pump at a rate of 25 µL/min. At least three videos lasting 30 seconds documenting Brownian motion of nanoparticles were recorded and at least 1000 completed tracks were analyzed by NanoSight software (NTA 2.3.5). Particles of 30-150 nm were designated as EVs.

### Extracellular vesicles uptake

EVs isolated from psoriasis-like HaCaT cells model were labeled with the green fluorescent dye PKH67 (Sigma). Differentiated THP-1 macrophages were then incubated with EVs (10^10^/mL) for 1 h (with or without 10 µM cytochalasin D pre-Hirsova et al. treatment), and their cellular internalization was observed using an FV10-MCPSU confocal microscope (Olympus, Janpan). EVs isolated from psoriasis-like mouse primary epidermis cells were labeled with the green fluorescent dye PKH67 and injected into the back of IMQ-induced psoriasis-like mice by microneedles. The effect of EVs on the pathogenesis of psoriasis was observed by mouse infrared imager (AniView SE, Guangzhou Biolight Biotechnology, China).

### Transient transfection

A siRNA (small interfering RNA) was used to silence TGFβR1 (si-TGFβ-R1, 5′- AGAAGUUGCUGUUAAGAUAdTdT-3′, designed and synthetized by Hanbio; Shanghai, China) in HaCaT cells. Cells transfected with scramble siRNA (5′- UUCUCCGAACGUGUCACGUdTdT-3′, Hanbio) were used as controls. Cells were grown in 60-mm dishes and transiently transfected with SmartPool siRNA (5 nM, Hanbio) using LipoFiter 3.0 (Hanbio). Experiments were performed 48 h after transfection. Then, the cells and culture supernatant were collected for the subsequent experiments. The same method was used to silence LRG1 (si-LRG1, 5′-GGCUACAUCUAGAAGGCAATT-3′, Hanbio) in Hacat cells. Cells transfected with scramble siRNA (5′-UUCUCCGAACGUGUCACGUTT -3′, Hanbio) were used as controls.

### Stable transfection

To establish a stable HaCaT-LRG1 overexpressing cell line, HaCaT cells were transducted with lentiviral RNAi vector (LRG1-gene, 5'-ATGTCCTCTTGGAGCAGACAGCGACCAAAAAGCCCAGGGGGCATTCAACCCCATGTTTCTAGAACTCTGTTCCTGCTGCTGCTGTTGGCAGCCTCAGCCTGGGGGGTCACCCTGAGCCCCAAAGACTGCCAGGTGTTCCGCTCAGACCATGGCAGCTCCATCTCCTGTCAACCACCTGCCGAAATCCCCGGCTACCTGCCAGCCGACACCGTGCACCTGGCCGTGGAATTCTTCAACCTGACCCACCTGCCAGCCAACCTCCTCCAGGGCGCCTCTAAGCTCCAAGAATTGCACCTCTCCAGCAATGGGCTGGAAAGCCTCTCGCCCGAATTCCTGCGGCCAGTGCCGCAGCTGAGGGTGCTGGATCTAACCCGAAACGCCCTGACCGGGCTGCCCCCGGGCCTCTTCCAGGCCTCAGCCACCCTGGACACCCTGGTATTGAAAGAAAACCAGCTGGAGGTCCTGGAGGTCTCGTGGCTACACGGCCTGAAAGCTCTGGGGCATCTGGACCTGTCTGGGAACCGCCTCCGGAAACTGCCCCCCGGGCTGCTGGCCAACTTCACCCTCCTGCGCACCCTTGACCTTGGGGAGAACCAGTTGGAGACCTTGCCACCTGACCTCCTGAGGGGTCCGCTGCAATTAGAACGGCTACATCTAGAAGGCAACAAATTGCAAGTACTGGGAAAAGATCTCCTCTTGCCGCAGCCGGACCTGCGCTACCTCTTCCTGAACGGCAACAAGCTGGCCAGGGTGGCAGCCGGTGCCTTCCAGGGCCTGCGGCAGCTGGACATGCTGGACCTCTCCAATAACTCACTGGCCAGCGTGCCCGAGGGGCTCTGGGCATCCCTAGGGCAGCCAAACTGGGACATGCGGGATGGCTTCGACATCTCCGGCAACCCCTGGATCTGTGACCAGAACCTGAGCGACCTCTATCGTTGGCTTCAGGCCCAAAAAGACAAGATGTTTTCCCAGAATGACACGCGCTGTGCTGGGCCTGAAGCCGTGAAGGGCCAGACGCTCCTGGCAGTGGCCAAGTCCCAGTGA-3', Hanbio) at an MOI of approximately 20 in the presence of 2 µg/mL polybrene. After 24 h, culture medium was replaced with fresh medium in HaCaT cells. 72 h after transduction, puromycin was added to medium at the concentration of 1 mg/mL for stable cell line selection. The empty lentivector lenti-puromycin was used as negative control. After puromycin selection for 2 weeks, stable overexpressing LRG1-gene cells were obtained. Cells were harvested and the LRG1-gene expression level was determined by real time-qPCR.

### Adeno-associated virus transfection

Female BALB/c mice weighing 18 to 20 g each were used in the study. After 1 week of adaptive feeding, the BALB/c mice received 1mL/100g (4g chloral hydrate, diluted by normal saline to 100 mL) of peritoneal anesthesia. The shaved 2.5 cm × 2.5 cm skin on the center of the back was selected as the injection area, five points were injected with K14-AAV9-si-LRG1 (titer:1 x 10^11^), K14-AAV9-si-NC (1 x 10^11^), and NS. Intradermal injection, 50 uL per point, the injection was grid-like, each injection point was about 0.5 cm apart, the injection depth was 1-2 mm. The needle was removed and the wounds were pressed with sterile cotton balls to prevent the venom from leaking.

### CCK8 assay

HaCaT cells were seeded in 96-well plates, then cells were cultured in 100 μl serum-free basal medium. After HaCaT cells were adhered to the wall, differentiated THP-1 macrophages conditioned supernatant was added for co-culture, and the cell proliferation was evaluated using the CCK8 (Cell Counting Kit-8, BesBio; Shanghai, China) after 24 h of co-culture. Briefly, 10 µL CCK8 solution was added to each well, and cells were incubated for 3 h at 37℃, 5% carbon dioxide. Cell viability was detected at 450 nm.

### Real-time reverse transcriptase-PCR

Total RNA of skin tissues and cells was isolated using TRIzol reagent (Invitrogen) and subsequent chloroform-isopropanol-ethanol purification, a NanoDrop spectrophotometer (NanoDrop One/OneC, Thermo Fisher Scientific) was used for RNA quality control. mRNA was reverse-transcribed with the PrimeScript RT reagent Kit with gDNA Eraser (TaKaRa Biotech Co.) and each test consumed 1 µg of total RNA according to the manufacturer's instructions. qPCR was carried out with the SYBR Premix Ex Taq II (TliRNaseH Plus) (TaKaRa Biotech Co.) using a LightCycler 96 (Roche) thermocycler. The mRNA expression levels of the related genes were normalized to that of the housekeeping gene β-actin. Relative expression levels were calculated according to the standard 2-^ΔΔCt^ method. The forward and reverse primers used for PCR are listed in Table S1.

### Western blot analysis

Cells or skin samples were lysed in RIPA (radio immunoprecipitation assay) buffer supplemented with protease and phosphatase inhibitor (Beyotime). Whole extracts were separated by 12 or 15% SDS-PAGE (sodium dodecyl sulfate polyacrylamide gel electrophoresis), transferred to a polyvinylidene difluoride membrane, and incubated with primary antibodies against LRG1/TSG101/ARF6/CD63/Calnexin/β-actin. The membranes were then washed in TBS-Tween 20 and incubated with the corresponding secondary antibodies. After extensive washing in TBS-Tween 20, protein bands were visualized with an ECL chemiluminescent kit (BeyoECL plus; Beyotime Biotechnology, Shanghai, China).

### ELISA

The cell culture supernatants were collected for cytokine evaluation and they were measured by the ELISA kits according to the manufacturer's instructions. Human LRG1 ELISA Kit was purchased from Elabscience (Wuhan, China). Human TNF-α, IL-4, IL-10 ELISA Kit were purchased from MultiSciences (Hangzhou, China). Human Monocyte Chemoattractant Protein (MCP-1), IL-23A and IL-8 ELISA Kit were purchased from ColorfulGene Biological Technology Co.,Ltd.(Wuhan, China). Human IL-1β ELISA Kit was purchased from Servicebio (Wuhan, China).

### Skin histopathology

Mouse skin tissues were fixed in 4% paraformaldehyde and embedded in paraffin. Sections (4 µm) were processed for histological examination according to a conventional method, and stained with H&E (Hematoxylin and eosin), Ki-67, F4/80, IL-23A, IL-17A and LRG1.The slides were scored in a blinded manner and deidentified.

### Statistical analysis

Data are expressed as the means ± SEM and represent at least three independent experiments. Statistical significance (*P < 0.05, **P < 0.01, ***P < 0.001) between two groups were assessed using the two-tailed unpaired Student's t-test. Differences between multiple groups were compared using one-way analysis of variance (ANOVA) followed by Student's t-test. Values of P < 0.05 were considered statistically significant. All analyses were performed using GraphPad Prism 9.0 software (GraphPad Software, La Jolla, CA, USA).

## Results

### Macrophages participate in promoting psoriasiform dermatitis

**Figure 1:**
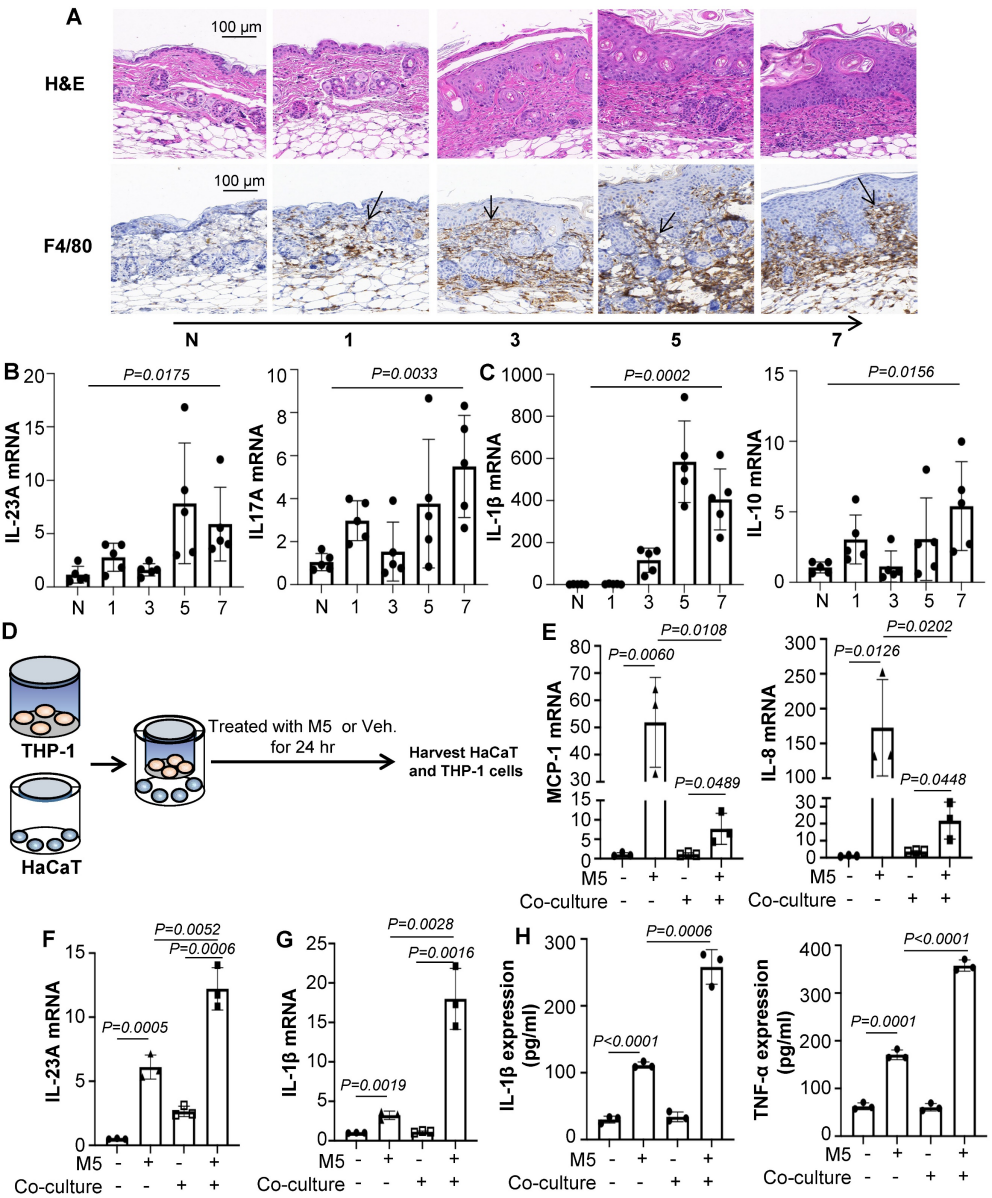
** Macrophages participate in psoriasiform dermatitis. (A)** Mice skin tissues stained with H&E and F4/80. Scale bar, 100 μm.** (B)** IL-23A, IL-17A mRNA was assessed by quantitative real-time PCR. **(C)** IL-1β, IL-10 mRNA was assessed by quantitative real-time PCR. **(D)** HaCaT and THP-1 co-culture system. THP-1 cells were differentiated using PMA pre-treatment for 24 h.** (E)** The level of MCP-1, IL-8 in HaCaT cells analysed by quantitative real-time PCR.** (F)** The level of IL-23A in HaCaT cells was analysed by quantitative real-time PCR.** (G)** The level of IL-1β in THP-1 cells was analysed by quantitative real-time PCR.** (H)** The protein levels of IL-1β and TNF-α in the co-culture supernatant were detected by ELISA. Similar results were obtained in 3 independent experiments with 5 mice per group or in triplicate culture assays.

Macrophage infiltration is a common feature of psoriasis and is believed to promote psoriasiform dermatitis. A prominent accumulation of F4/80^+^ macrophages was seen in IMQ-treated mice along with the increased treatment time (Figure 1A). Consistent with the severity of skin lesions seen on H&E and Ki-67-stained sections (Figure 1A and Figure S1A). In addition, IMQ-treatment substantially increased IL-23A and IL-17A expression (Figure 1B). These infiltrates were accompanied by up-regulation of the inflammatory cytokines IL-1β and IL-10 at the mRNA level (Figure 1C). And we also found that the expression of IL-22, IL-6, TGF-β and CD86 were enhanced, while TNF-α and Arg1 were no obviously change (Figure S1B).

To explore the effects of macrophages promoting psoriasiform dermatitis *in vitro*, we firstly employed a co-culture system as shown schematically in Figure 1D. The human monocytic cell line (THP-1), which can be differentiated into macrophage-like cells, in the upper chamber of a trans-well plate were co-cultured with HaCaT in the lower chamber. The co-culture system was treated with M5 (TNF-α, IL-17A, OSM, IL-1α, IL-22, 10ng/mL) for 24 hours and harvested for analysis. As shown in Figure 1E and 1F, co-cultured with THP-1 induced significantly down-regulated levels of MCP-1 and IL-8 in the HaCaT compared to only treated with M5, while up-regulated the expression level of IL-23A. ELISA results were also showed the same change (Figure S1C). Additionally, IL-1β, CD86 and IL-10 expression level were increased in the co-culture THP-1 with M5 treated, although there was no significant change in TNF-α and IL-4 (Figure 1G and Figure S2A). But detected the IL-1β and TNF-α expression levels in the co-culture media by ELISA, TNF-α expression level was obviously increased (Figure 1H). These data indicated that macrophages participate in promoting psoriasiform dermatitis and may be mediated by intercellular communication with keratinocyte.

### Supernatant of HaCaT cells stimulated by M5 promotes M1 polarization

**Figure 2:**
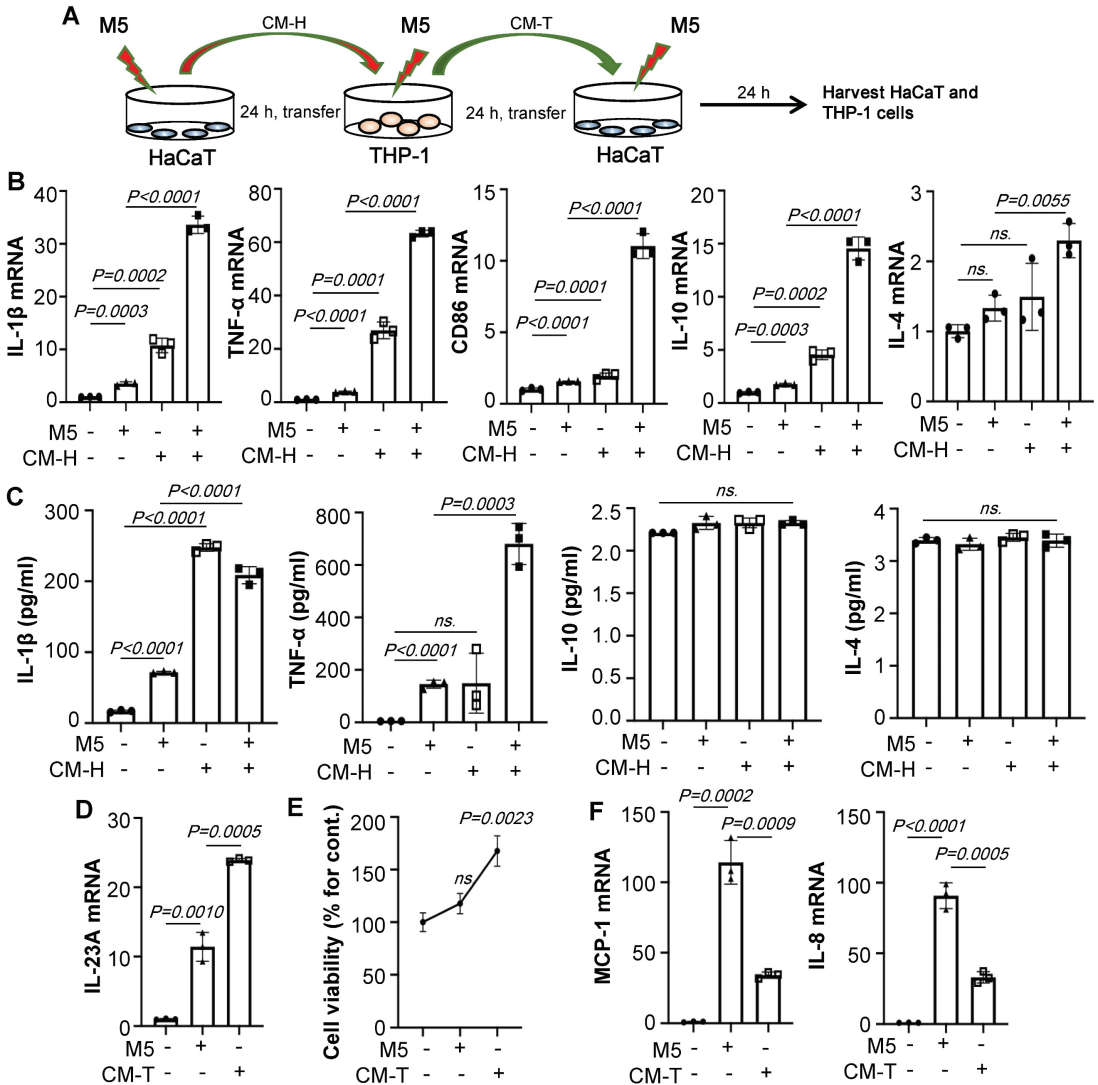
** M5-stimulated Keratinocytes induce macrophage polarization. (A)** HaCaT/THP-1 conditioned media culture model. THP-1 cells were differentiated by PMA pre-treatment for 24 h. **(B)** The mRNA levels of IL-1β, TNF-α, CD86, IL-10, IL-4 in THP-1 cells analysed by quantitative real-time PCR. **(C)** The protein levels of IL-1β, TNF-α, IL-10, IL-4 in THP-1 cell supernatant were detected by ELISA. **(D)** The mRNA level of IL-23A in HaCaT cells analysed by quantitative real-time PCR. **(E)** Cell viability detected by CCK-8 kit. **(F)** The mRNA levels of MCP-1, IL-8 in HaCaT cells analysed by quantitative real-time PCR. Similar results were obtained in 3 independent experiments or in triplicate culture assays.

To determine whether the polarization of macrophages is more affected by the supernatant of keratinocytes than by M5 alone, HaCaT/THP-1 conditioned media culture model was established (Figure 2A). As shown in Figure 2B, conditioned media from M5-treated HaCaT significantly increased the IL-1β, TNF-α, CD86, IL-10, and IL-4 expression of THP-1 cells compared to only those treated with M5. Furthermore, ELISA results showed that conditioned media from M5-treated HaCaT promoted IL-1β and TNF-α secretion, but there was no significant change in IL-10 and IL-4, indicating that the supernatant of HaCaT stimulated by M5 promotes the polarization of macrophages towards M1 (Figure 2C). In addition, conditioned media of THP-1 cells (CMT) also up-regulates the expression of IL-23A (Figure 2D). Interestingly, a dose of M5 did not induce HaCaT obviously proliferation, but the level of cell viability was increased with the addition of the M5-CMT-induced group compared to M5-induced groups (Figure 2E). Similar to co-culture results, CMT induced significantly down-regulated levels of MCP-1 and IL-8 in the HaCaT (Figure 2F). The above results indicated that keratinocyte-induced promotes macrophage M1 polarization.

### Keratinocyte-derived EV promotes psoriasiform dermatitis by recruiting and promoting M1 macrophage polarization

**Figure 3:**
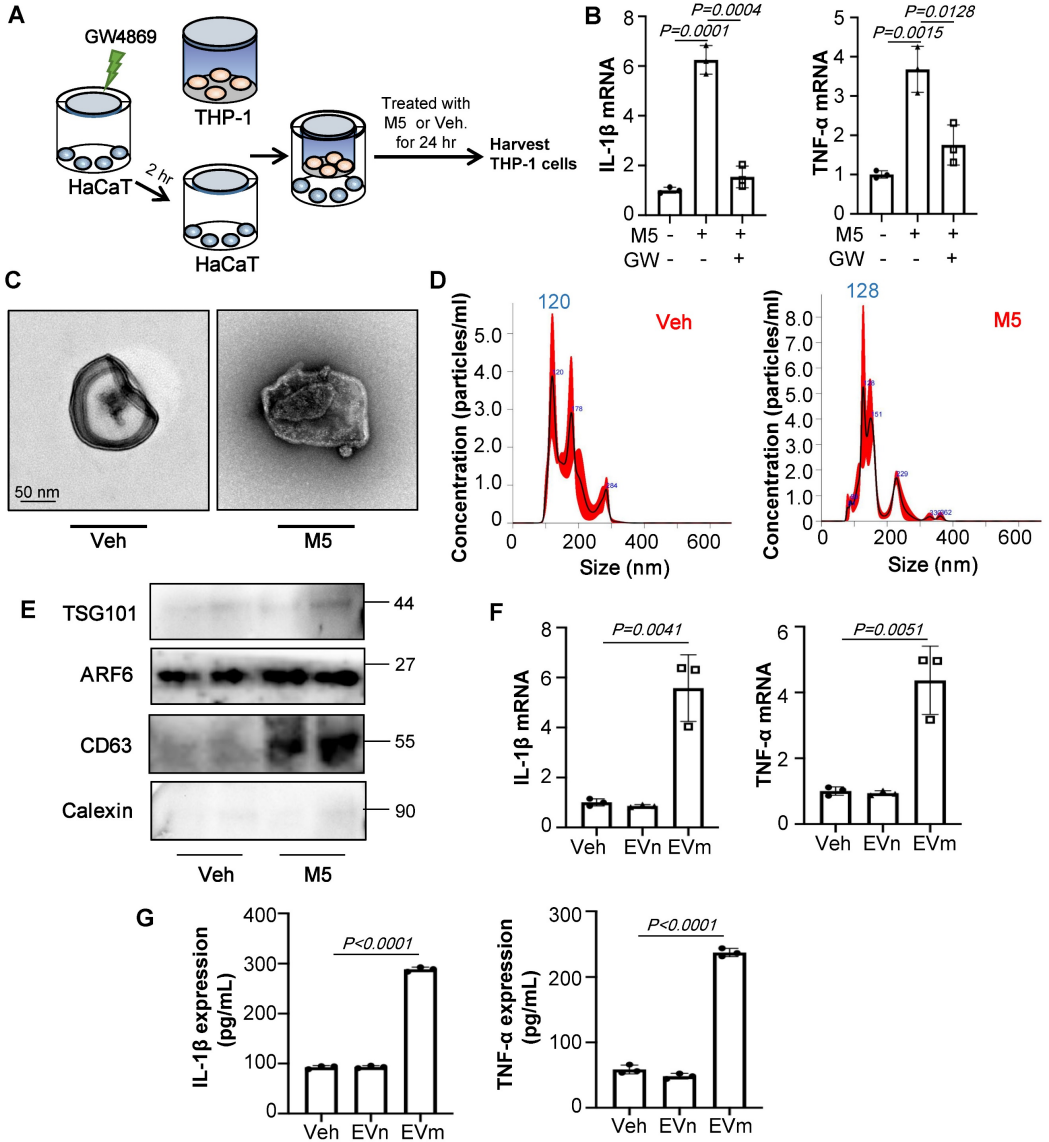
** Keratinocyte promotes macrophage polarization by secrete EVs. (A)** GW4869 pre-treated HaCaT and THP-1 co-culture system. THP-1 cells were differentiated using PMA pre-treatment for 24 h.** (B)** The level of IL-1β, TNF-α in THP-1 cells analysed by quantitative real-time PCR.** (C)** Transmission electron photomicrographs of HaCaT-derived EV.** (D)** HaCaT-derived EV representative image with nanoparticle tracking analysis (NTA). **(E)** Expression of TSG101, ARF6, CD63 and Calexin in HaCaT-derived EV were detected by western blot.** (F)** The level of IL-1β, TNF-α in THP-1 cells treated with HaCaT-derived EV analysed by quantitative real-time PCR.** (G)** The protein levels of IL-1β and TNF-α in THP-1 cell supernatant were detected by ELISA. Similar results were obtained in 3 independent experiments or in triplicate culture assays.

Cell-to-cell communication via direct contact or through soluble factors is of vital importance for multicellular organisms. To test whether EVs were involved in the communication of keratinocyte with macrophages, we used GW4869, a known inhibitor of EV secretion, to treat keratinocyte (Figure 3A). As shown in Figure 3B, GW4869 pre-treated HaCaT group significantly decreased the IL-1β and TNF-α expression of THP-1 cells compared to group treated with only M5. To confirm the effect of EVs *in vitro*, we incubated THP-1 with the purified EVs released from M5-treated HaCaT (10^10^/mL). First, we test the size characteristics of the released EVs by electron microscopy (Figure 3C). Then quantified via NTA (Figure 3D) and test the exosomal markers by immunoblot analysis (Figure 3E). Similar to results *in vivo*, RT-qPCR showed that EVs released from M5-treated HaCaT significantly promoted IL-1β and TNF-α expression of THP-1 cells (Figure 3F). And ELISA results were also showed the same change (Figure 3G).

**Figure 4:**
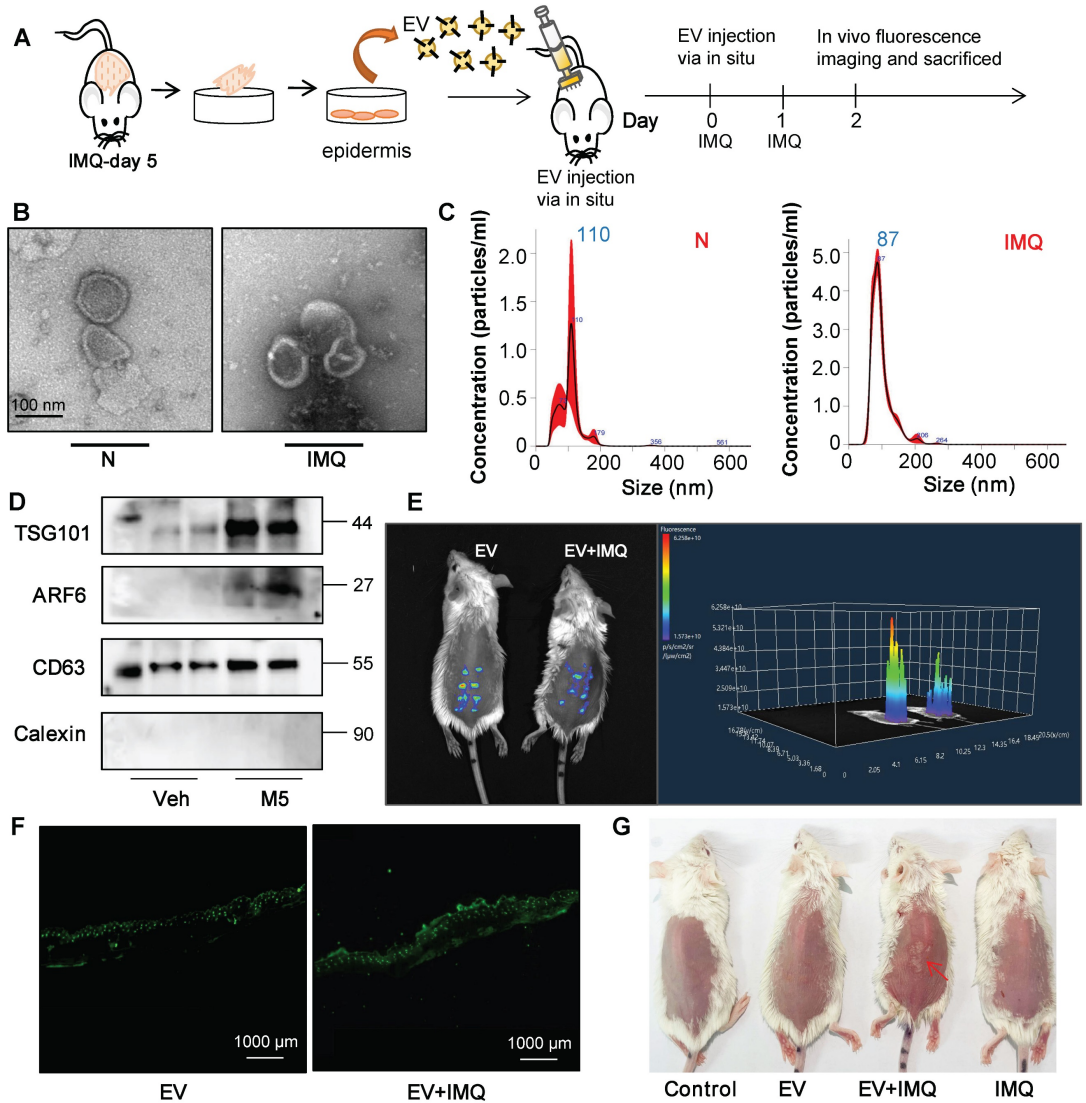
** Keratinocyte-derived EVs promote IMQ-induced psoriasiform dermatitis. (A)** Purified EVs were isolated from primary keratinocyte of 5-day IMQ-induced mice and PKH67-labelled EVs were injected into mice via skin *in situ* injection.** (B)** Transmission electron photomicrographs of primary keratinocytes-derived EV of IMQ-induced mice.** (C)** Primary keratinocytes-derived EV of IMQ-induced mice representative image with nanoparticle tracking analysis (NTA). **(D)** Expression of TSG101, ARF6, CD63 and Calexin in primary keratinocytes-derived EV of IMQ-induced mice were detected by western blot.** (E)** Imaging of PKH67-labeled EVs in mice. About 100 μg (at protein level) in 100 μL EV from IMQ exposured mice primary keratinocytes, labeled with PKH67 were injected via *in situ*. About 48 h after the *in situ* injection, *in vivo* fluorescence imaging were performed.** (F)** Fluorescence imaging were performed in skin tissue section. Scale bar, 1000 μm.** (G)** Photos of skin lesions on the back of mice. Similar results were obtained in 3 independent experiments with 3 mice per group or in triplicate culture assays.

**Figure 5:**
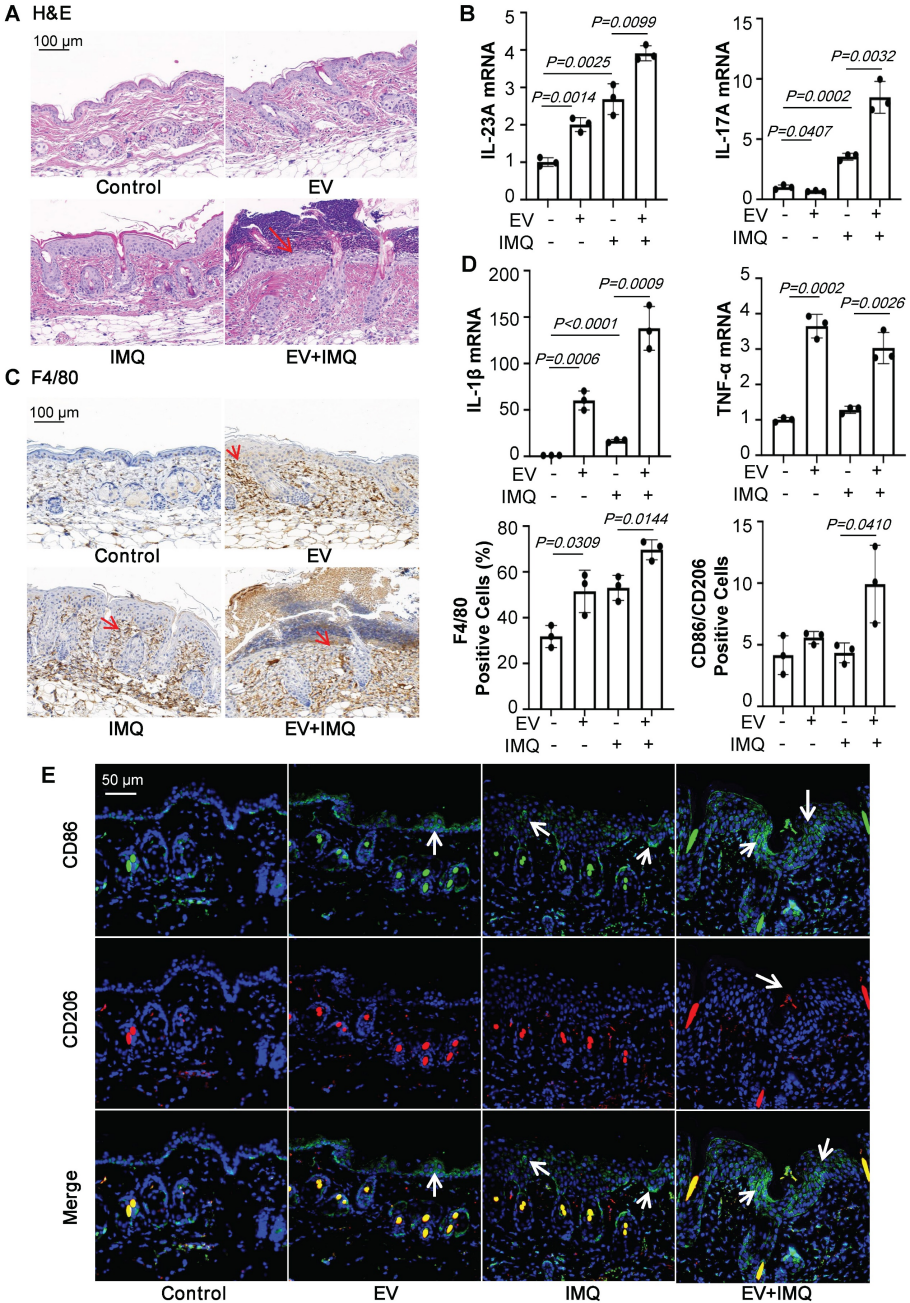
** Keratinocyte-derived EVs promote M1 macrophage polarization and psoriasiform dermatitis**. **(A)** Mice skin tissues stained with H&E. Scale bar, 100 μm. **(B)** IL-23A, IL-17A mRNA was assessed by quantitative real-time PCR. **(C)** Mice skin tissues stained with F4/80. Scale bar, 100 μm. **(D)** IL-1β, TNF-α mRNA was assessed by quantitative real-time PCR. **(E)** Mice skin tissues stained with CD86 and CD206. Scale bar, 50 μm. Similar results were obtained in 3 independent experiments with 3 mice per group.

To further certify that keratinocyte promotes macrophage polarization and accelerates skin lesions by releasing EVs, purified EVs were isolated from the epidermis of 5-day IMQ-induced psoriasiform dermatitis model mice and fluorescence-labelled the EV with PKH67. And PKH67-labeled EVs were injected into mice, treated with IMQ or vehicle for 2 days, via skin *in situ* injection (Figure 4A). Released EVs were isolated from epidermis cells culture media and the size characteristics were confirmed by electron microscopy (Figure 4B), and quantified via nanoparticle tracking analysis (NTA). Over a 5-day stimulation period, IMQ-induced psoriasiform dermatitis model mice increase approximately 2.5-fold in the release of EVs in keratinocyte cells (Figure 4C). Immunoblot analysis indicated that the isolated EV contained the established exosomal and microvesicular markers such as tumor susceptibility gene 101 (TSG101), adenosine diphosphate ribosyltion factor 6 (ARF6), CD63 and Calexin (Figure 4D). It was found that fluorescence-labeled epidermis cell-derived EVs were most localized in the epidermis (Figure 4E and 4F). Most importantly, EVs of epidermis induced with IMQ for 5-day promoted psoriasiform dermatitis in IMQ-induced 2-day mice (Figure 4G). H&E and Ki-67 staining also further revealed that these EVs enhanced skin lesions and proliferation in IMQ-induced 2-day mice (Figure 5A and Figure S2B). And EVs-treatment substantially increased IL-23A and IL-17A expression (Figure 5B and Figure S2C). Interestingly, IMQ-induced 5-day, epidermis-derived EV could increase the recruiting of macrophage in mice with or without IMQ treatment (Figure 5C). These infiltrates were accompanied by up-regulation of the inflammatory cytokines IL-1β and TNF-α at the mRNA level (Figure 5D). In addition, we have detected the CD86 and CD206 expression of macrophage by immunofluorescence, and analyzed the ratio of these two markers in the EV-treated skin (Figure 5E).

The above data suggests that keratinocytes-derived EVs promote IMQ-induced psoriasiform dermatitis through recruiting and activating macrophages.

### LRG1-enriched EVs from Keratinocytes mediated macrophage polarization via TGFβR1

We first examined whether phagocytosis was required for macrophage polarization by EVs. It was demonstrated that the level of inflammation cytokines was no change with the addition of Cytochalasin D, which is a pharmacologic inhibitor of phagocytosis, suggesting that Cytochalasin D efficiently inhibited phagocytosis of fluorescently labeled EVs but had no effect on M5-treated, HaCaT-derived EVs induced inflammation (Figure S3A-B).

**Figure 6:**
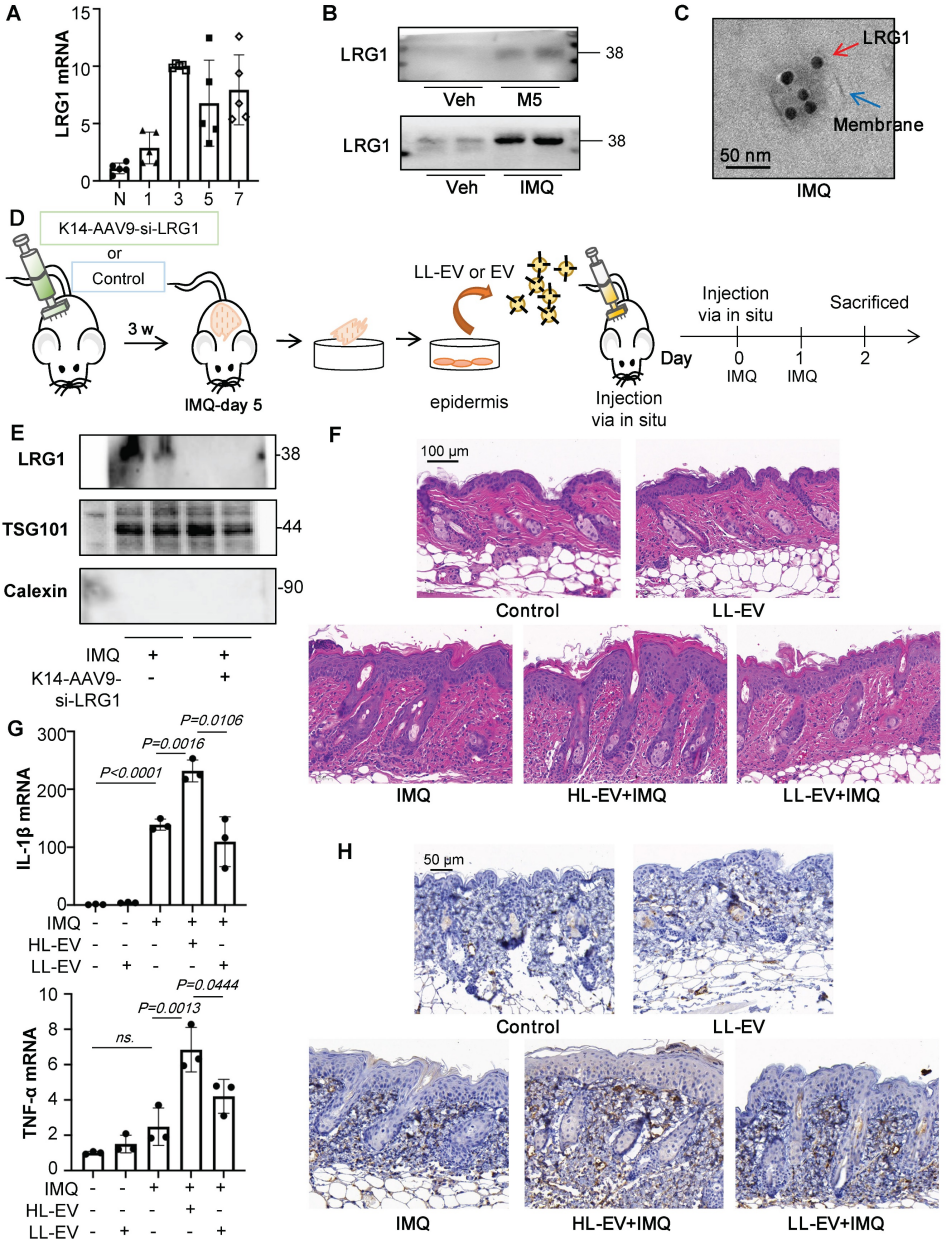
** LRG1-enriched EVs promote M1 macrophage polarization and psoriasiform dermatitis**. **(A)** The level of LRG1 in IMQ-induced mouse model analysed by quantitative real-time PCR. **(B)** Expression of LRG1 in HaCaT-derived EV and primary keratinocytes-derived EV were detected by western blot. **(C)** Representative transmission electron photomicrographs immunogold- labeled with an anti-LRG1 antibody of primary keratinocytes-derived EV. Scale bar: 50 nm. **(D)** The AAV9 vectors delivering si-LRG1 driven by Keratin 14 (K14) promoter induce to knockdown the expression of LRG1 of IMQ-treated mice by skin *in situ* injection, released EVs were isolated from primary epidermis cells culture. **(E)** Expression of TSG101, LRG1 and Calexin in primary keratinocytes-derived EV of IMQ-induced mice were detected by western blot. **(F)** Mice skin tissues stained with H&E. Scale bar, 100 μm. **(G)** IL-1β, TNF-α mRNA was assessed by quantitative real-time qPCR. **(H)** Mice skin tissues stained with F4/80. Scale bar, 50 μm. Similar results were obtained in 3 independent experiments with 3 mice per group.

Our previous study revealed that LRG1-enriched EVs could mediate macrophage polarization 14. We also found that LRG1 expression was up-regulated in M5-induced keratinocytes of co-culture and conditional medium system by RT-qPCR, but down-regulated while existing THP-1 (Figure S3C). Consistent with *in vitro* data, there was obviously up-regulation of LRG1 in IMQ-treated mice (Figure 6A and Figure S3D). Interestingly, we isolated EV from primary keratinocytes from the IMQ model and found the protein level of LRG1 was significantly increased in the IMQ group (Figure 6B), which was also confirmed with immunogold labeling and electron microscopy in Figure 6C.

EV from primary keratinocytes from the IMQ-treated mice promote IMQ-induced psoriasiform dermatitis, but it is currently unclear whether LRG1 expression in EVs is a major factor. Therefore, we used EVs derived from keratinocytes with downregulated LRG1 expression under the same conditions for comparison *in vivo*. Recombinant AAV (rAAV) system has been proved to be safe for therapeutic payload delivery, we chose AAV9 vectors delivering si-LRG1 driven by Keratin 14 (K14) promoter induce to knockdown the expression of LRG1 of IMQ-treated mice by skin *in situ* injection, released EVs were isolated from primary epidermis cells culture (Figure 6D). A significant decrease of LRG1 protein level was seen in the EV from K14-AAV9-si-LRG1 treated group (Figure 6E). H&E staining revealed that these Low-LRG1-enriched EVs (LL-EVs) alleviated skin lesions in IMQ-induced mice, compared to the High-LRG1-enriched EVs (HL-EVs) group (Figure 6F). Consistent with decreased pro-inflammatory cytokine production following EV with Low expression of LRG1 (LL-EV) injection (Figure 6G), macrophage infiltration was measured by immunohistochemistry analysis (Figure 6H).

**Figure 7:**
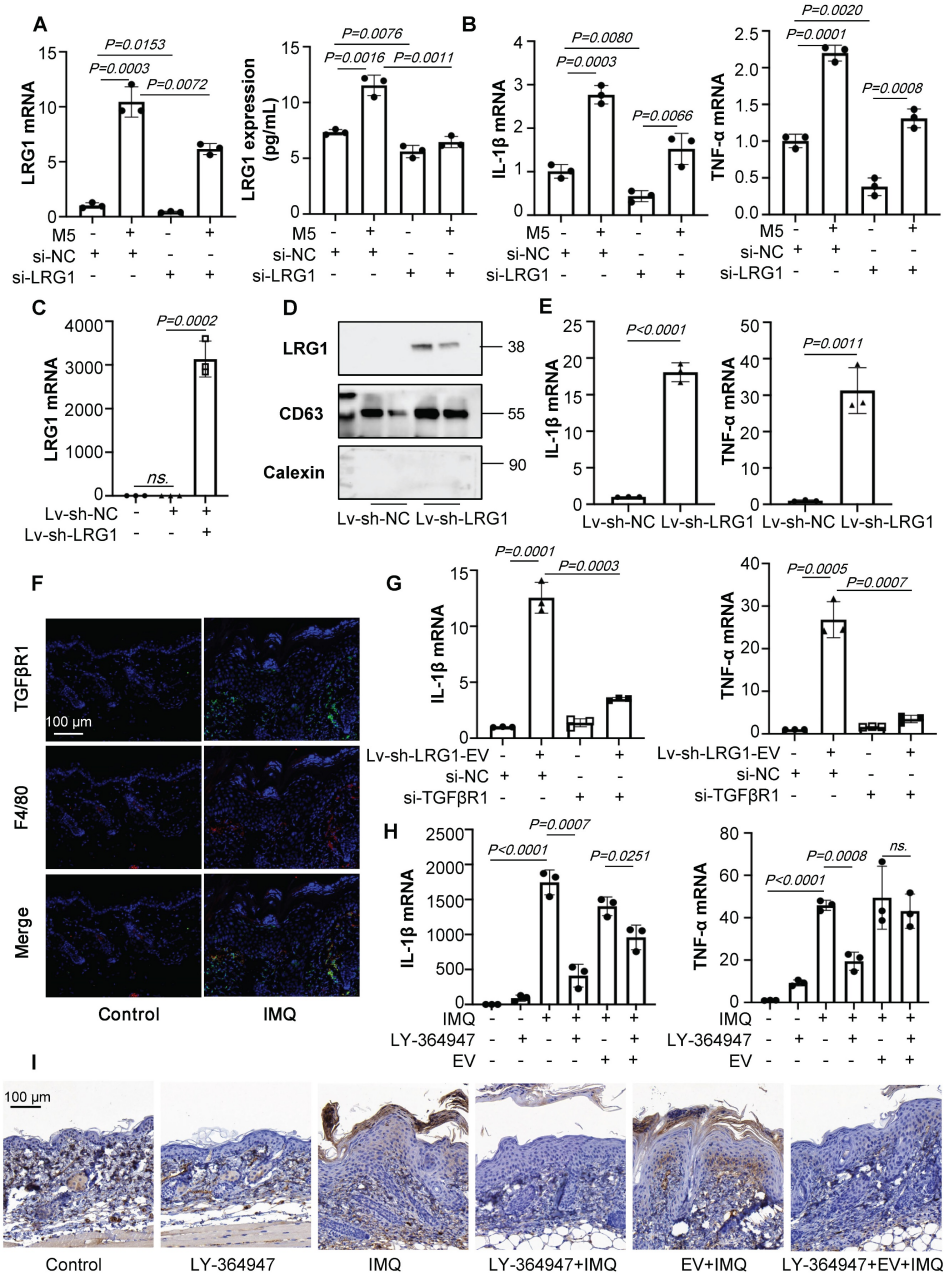
** LRG1-enriched EVs from Keratinocytes mediate macrophage polarization via TGFβR1. (A)** The level of LRG1 in HaCaT cells treated with si-LRG1 analysed by quantitative real-time PCR and ELISA.** (B)** The mRNA levels of IL-1β, TNF-α in THP-1 cells treated with conditional medium from si-LRG1-stimulated HaCaT cells analysed by quantitative real-time PCR.** (C)** The level of LRG1 in HaCaT cells treated with Lv-sh-LRG1 analysed by quantitative real-time PCR.** (D)** The level of LRG1 in EV derived from HaCaT treated with Lv-sh-LRG1 detected by western blot.** (E)** The mRNA levels of IL-1β, TNF-α in THP-1 cells treated with EV derived from Lv-sh-LRG1-stimulated HaCaT cells analysed by quantitative real-time PCR.** (F)** Mice skin tissues stained with TGFβR1. Scale bar, 100 μm.** (G)** The mRNA levels of IL-1β, TNF-α in si-TGFβR1 pre-treated THP-1 cells treated with EV derived from Lv-sh-LRG1-stimulated HaCaT cells analysed by quantitative real-time PCR.** (H)** IL-1β, TNF-α mRNA was assessed by quantitative real-time PCR.** (I)** Mice skin tissues stained with F4/80. Scale bar, 100 μm. Similar results were obtained in 3 independent experiments with 3 mice per group or in triplicate culture assays.

To further explore the role of keratinocytes-derived LRG1 on macrophage polarization *in vitro*, we treated HaCaT cells with siRNA-LRG1. siRNA-LRG1-pretreated, M5-induced HaCaT cells expressed much lower levels of LRG1 than M5 treated group (Figure 7A), then in different conditional medium treated THP-1 cells, we found that down-regulation of LRG1 in HaCaT could decrease the expression of IL-1β and TNF-α (Figure 7B). Furthermore, we treated HaCaT cells with lentiviral RNAi vector to up-regulate the expression of LRG1, and purified EVs were isolated from HaCaT cells with LRG1 overexpression (Figure 7C and 7D). Interestingly, we incubated THP-1 with purified EVs released from HaCaT cells with LRG1 overexpression (10^10^/mL), and RT-qPCR results showed that EVs released from Lv-sh-LRG1-HaCaT significantly up-regulated IL-1β and TNF-α expression of THP-1 cells compared with control group (Figure 7E).

Previous study has showed that LRG1 could regulate the inflammatory cytokines expression via the TGFβR1. However, it is unknown whether keratinocytes-derived, LRG1-enriched EVs could promote macrophage polarization via TGFβR1. Interestingly, we found that TGFβR1 expression of F4/80^+^ macrophages were significantly increased after IMQ treated (Figure 7F).

To determine whether TGFβR1 mediated LRG1-enriched EV-induced polarization of macrophages, we then treated the THP-1 cells with siRNA-TGFβR1 for 24 hours (Figure S4A). RT-PCR analysis indicated that the knockdown of TGFβR1 had significantly inhibited the expression of IL-1β and TNF-α (Figure 7G).

To address the role of TGFβR1 in the pathogenesis of IMQ-induced mice, skin lesions, macrophage infiltration and keratinocytes proliferation was measured in animals treated with IMQ in the presence or absence of TGFβR1 inhibitors. Different concentrations of A83-01 and LY-364947 were used to downregulate the expression of TGFβR1 in IMQ-induced mice by skin *in situ* injection (Figure S4B-C). Then we choose the LY-364947 (1 mg/kg) for the following experiment. H&E staining revealed that the LY-364947 could alleviated skin lesions in IMQ-induced mice (Figure S4D). The expression of IL-1β and TNF-α were increased after IMQ treatment, but these increases were significantly blunted in LY-364947 administration group mice (Figures 7H). As expected, downregulation of TGFβR1 had significantly inhibit the macrophage infiltration and alleviate the IMQ-induced psoriasiform dermatitis (Figures 7I).

Taken together, keratinocyte-derived, LRG1-enriched EVs promoted polarization of macrophages in a TGFβR1-dependent manner.

## Discussion

Psoriasis patients have an increased level of circulating monocytes in peripheral blood 15. Leite et al. recent studies have found that removing macrophages could improve the severity of psoriasis, indicating that the infiltration and activation of macrophages play an important role in the onset of psoriasis 4, 5. In the current study, we found that F4/80^+^ macrophage accumulation polarization in IMQ-treated mice in lesional skin accompanied with the increased stimulation time, which was consistent with previous report 8, 16. However, the regulatory mechanism of macrophages infiltration and polarization in psoriasis lesions has not been clarified, and the correlation between macrophages and keratinocytes needs to be further explored.

Keratinocytes are the main constituents of the epidermis and contribute to the formation of psoriatic lesions. Furthermore, they actively affect the epidermal microenvironments and influence the functions of infiltrated immune cells, such as neutrophils, in the disease 2. We also found that macrophages could significantly promote keratinocyte inflammation *in vitro*, and co-cultured with THP-1 down-regulate MCP-1 and IL-8 in the HaCaT, which were monocyte attract cytokine. In addition, IL-1β, CD86, and IL-10 expression levels were evidently increased in the co-culture THP-1 with M5 treated, although there was no significant change in TNF-α and IL-4. Then we indicated that macrophage activation may be mediated by intercellular communication with keratinocytes. Interestingly, HaCaT/THP-1 conditioned media culture model results could indicate that the excretion of keratinocyte induced macrophage polarization.

Recent studies have revealed that EVs may participate in psoriasis lesions 11, 17. Keratinocytes also can secrete EVs, which could target melanocytes to modulate pigmentation 18. However, the role of keratinocyte EVs in mediating the communication between keratinocytes and macrophages is yet to be explored. In this study, we found that the IMQ-induced 5-day, epidermis-derived EVs promoted the infiltration of macrophage and enhanced the progression of skin lesions in IMQ-induced 2-day mice. Furthermore, we incubated THP-1 with purified EVs released from M5-treated HaCaT, similar to results *in vivo*, EVs released from M5-treated HaCaT significantly promoted IL-1β and TNF-α expression of THP-1 cells. *In vivo* and *in vitro* data suggest that keratinocyte-derived EVs promote macrophage infiltration and polarization in IMQ-induced psoriasiform dermatitis. Interestingly, EVs secreted by the overall psoriasis-like keratinocytes could promote the psoriasiform dermatitis, but the role of EVs from different states of keratinocytes, such as proliferating and differentiating is still unknown, which will become a focus of our future study.

In our previous study, we demonstrated that LRG1-enriched EV could mediate macrophage polarization 14. LRG1 is a multifunctional pathogenic signaling molecule, which belongs to secretory glycoprotein 19. After various inflammatory stimuli, including infection, injury, autoimmune diseases, and tumor-related inflammation, LRG1 may be produced in systemic or local tissues, including pro-inflammatory cytokines IL-6, TNF-α, and IL-1β may promote the production of LRG1 and directly participate in inflammatory diseases such as Lupus nephritis 20 and Rheumatoid arthritis 21. Studies have shown that persistently high levels of LRG1 can maintain an inflammatory response and promote disease progression through a wide range of biological functions 22. At present, it has been reported that LRG1 might be a useful biomarker to detect the occurrence of psoriasis in adults 23-25. The development of new biomarkers has an urgent clinical need for early diagnosis and management of certain inflammatory diseases, and LRG1 is regulated by various pro-inflammatory cytokines, making it a highly promising candidate drug target for development 26. However, the role of LRG1 in psoriasis remains largely unexplored. In our study, it was observed that the level of LRG1 was significantly increased in isolated EV derived from primary keratinocytes of IMQ-induced mice and HaCaT cell lines treated with M5 cytokines compared with control groups respectively. Interestingly, down-regulation of LRG1 inhibited macrophages toward a proinflammatory phenotype, while purified EVs derived from up-regulation of LRG1 HaCaT cells promoted the macrophages toward a proinflammatory phenotype. Similarly, EVs derived from primary keratinocytes with downregulated LRG1 were also inhibiting macrophages toward a proinflammatory phenotype. All the above data supported the conclusion that keratinocyte-derived, LRG1-enriched EVs induced polarization of macrophages. Transforming growth factor (TGF)-β is a cytokine of the bone morphogenetic protein (BMP) activator family, which mediates the extensive role of the immune system 27. LRG1 can promote macrophage polarization by regulating the TGF-βR1 signaling pathway of endothelial cells 14. Herein, we found that keratinocytes-derived, LRG1-enriched EV-mediated macrophage polarization is dependent on TGFβR1 signaling.

**Figure 8:**
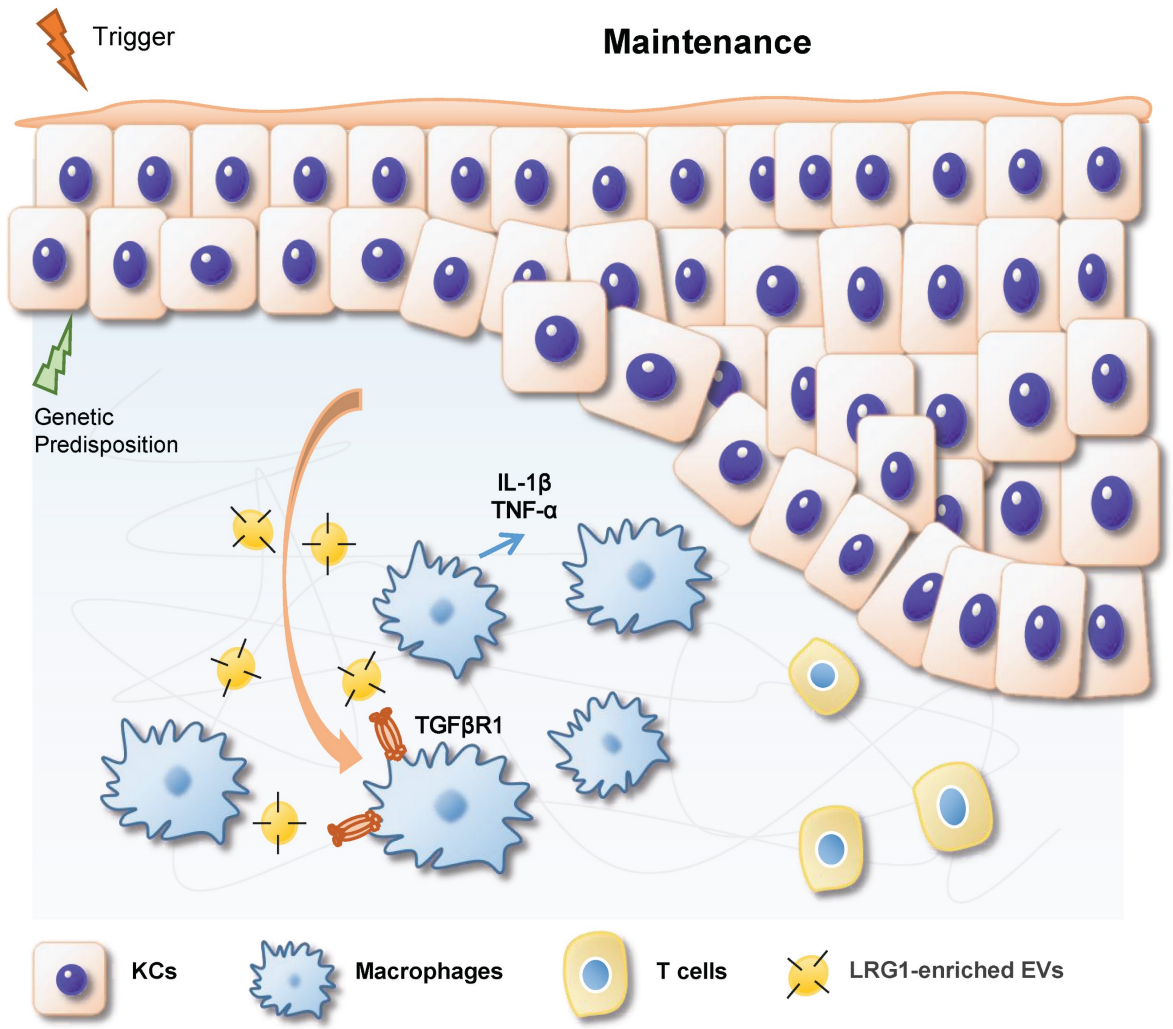
During IMQ-induced psoriasiform dermatitis progression, LRG1-enriched EVs released from keratinocytes regulates macrophage by a TGFβR1-dependent pathway and subsequently up-regulates expression of some inflammatory genes, thereby promoting psoriasiform dermatitis.

In summary, based on our findings, we propose a working model in Figure 8. During IMQ-induced psoriasiform dermatitis progression, LRG1-enriched EVs released from keratinocytes induce macrophage polarize to M1 by a TGFβR1-dependent pathway and subsequently up-regulates expression of some inflammatory genes, such as TNF-α, and IL-1β, thereby promoting psoriasiform dermatitis.

## Supplementary Material

Supplementary figures and table.

## Abbreviations

EVs: extracellular vesicles
IMQ: Imiquimod
LRG1: Leucine-rich α-2-glycoprotein 1
NTA: nanoparticle tracking analysis
TSG101: tumor susceptibility gene 101
ARF6: adenosine diphosphateribosyltion factor 6
TGFβR1: TGF beta Receptor 1
IgG: immunoglobulin G
HRP: horseradish peroxidase
EGF: Epidermal Growth Factor
BPE: Bovine Pituitary Extract
PMA: phorbol 12-myristate 13-acetate
THP-1: The human monocytic cell line
siRNA: small interfering RNA
MCP-1: Monocyte Chemoattractant Protein
CCK8: Cell Counting Kit-8
TNF-α: tumor necrosis factor α
IL: interleukin
Cyto D: Cytochalasin D
ANOVA: analysis of variance
BMP: bone morphogenetic protein
K14: Keratin 14
