# Supplementary material for: Keratinocyte-to-macrophage communication exacerbate psoriasiform dermatitis via LRG1-enriched extracellular vesicles
Source: Theranostics. 2024 Jan 1;14(3):1049–64. doi: 10.7150/thno.89180 (PMC10797285; doi:10.7150/thno.89180)
Supplement: Supplementary file 1 — Supplementary figures and table. [file thnov14p1049s1.pdf]

Supplementary Information Text

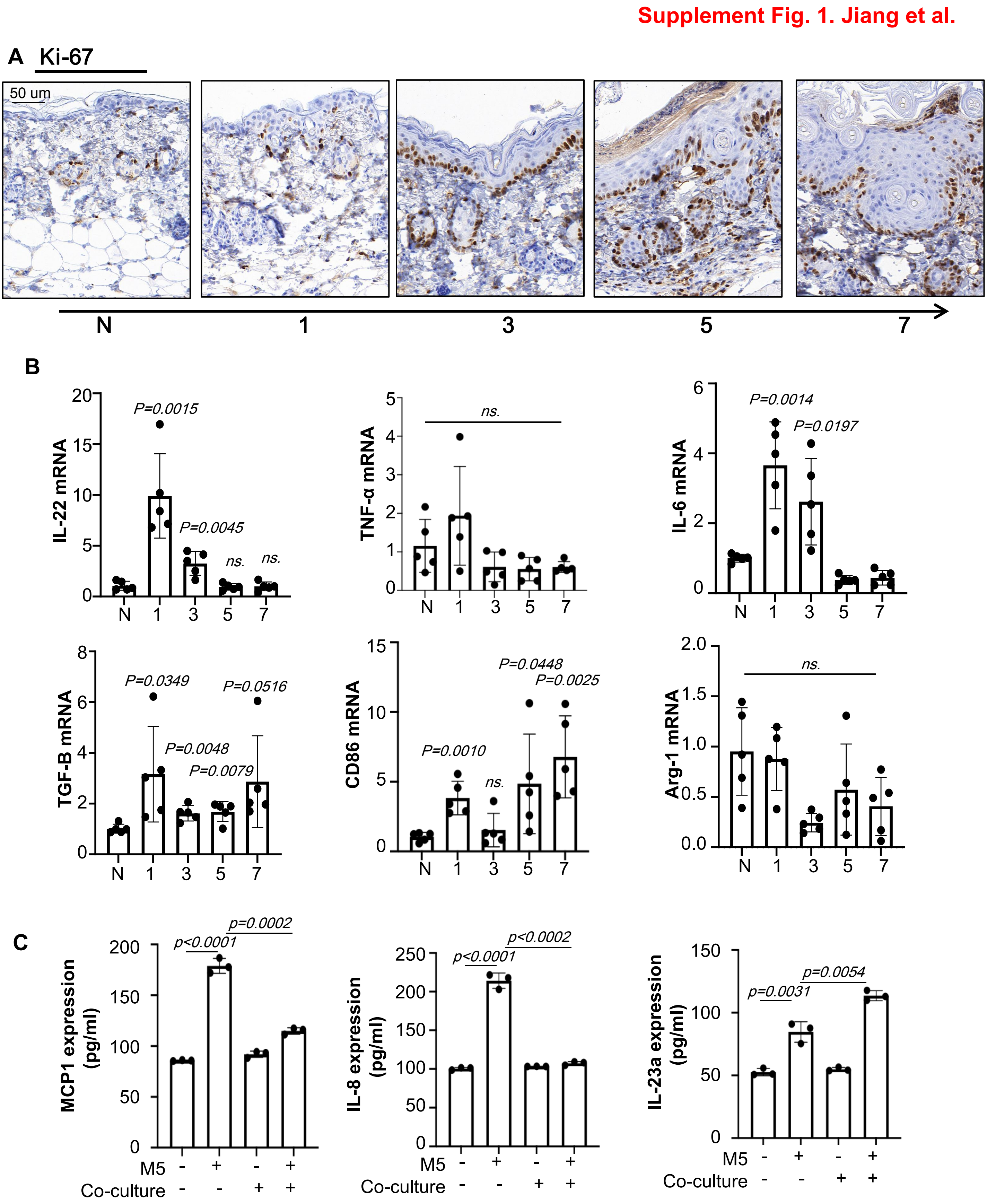

Fig. S1

(A) Mice skin tissues stained with Ki-67. Scale bar, 50 μM.
(B) IL-22, TNF-α, IL-6, TGF-B, CD86 and Arg1 mRNA was assessed by quantitative real-time PCR.
(C) The protein levels of MCP-1, IL-8, IL-23a in the co-culture supernatant were detected by ELISA.
Similar results were obtained in 3 independent experiments with 5 mice per group or in triplicate culture assays.

Supplement Fig. 2. Jiang et al.

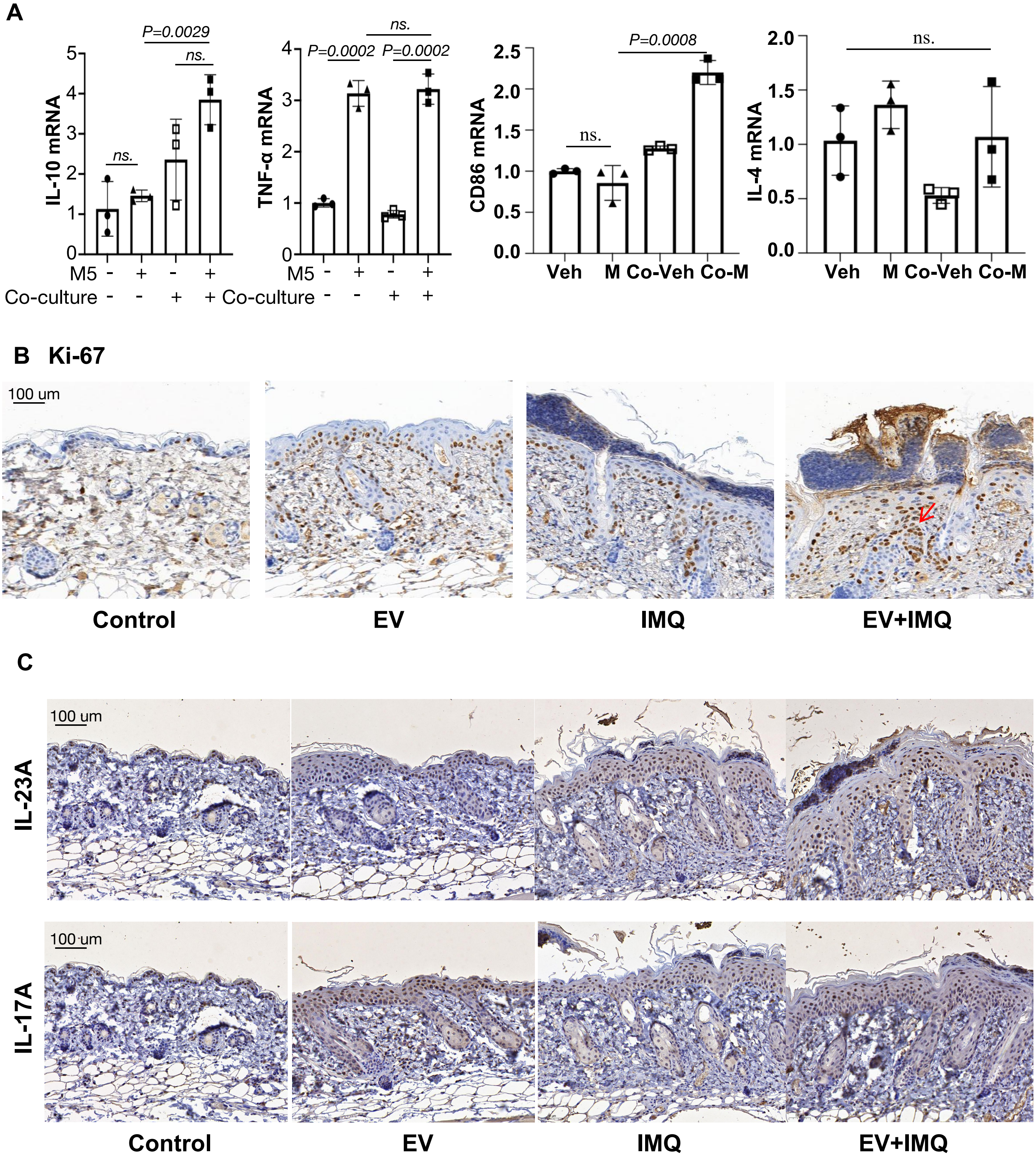

Fig. S2

(A) The mRNA levels of IL-10, TNF-α, CD86 and IL-4 in THP-1 cells analysed by quantitative real-time PCR.

(B) Mice skin tissues stained with Ki-67. Scale bar, 100 μm.

(C) Mice skin tissues stained with IL-23A and IL-17A. Scale bar, 100 μm.

Similar results were obtained in 3 independent experiments or in triplicate culture assays.

Supplement Fig. 3. Jiang et al.

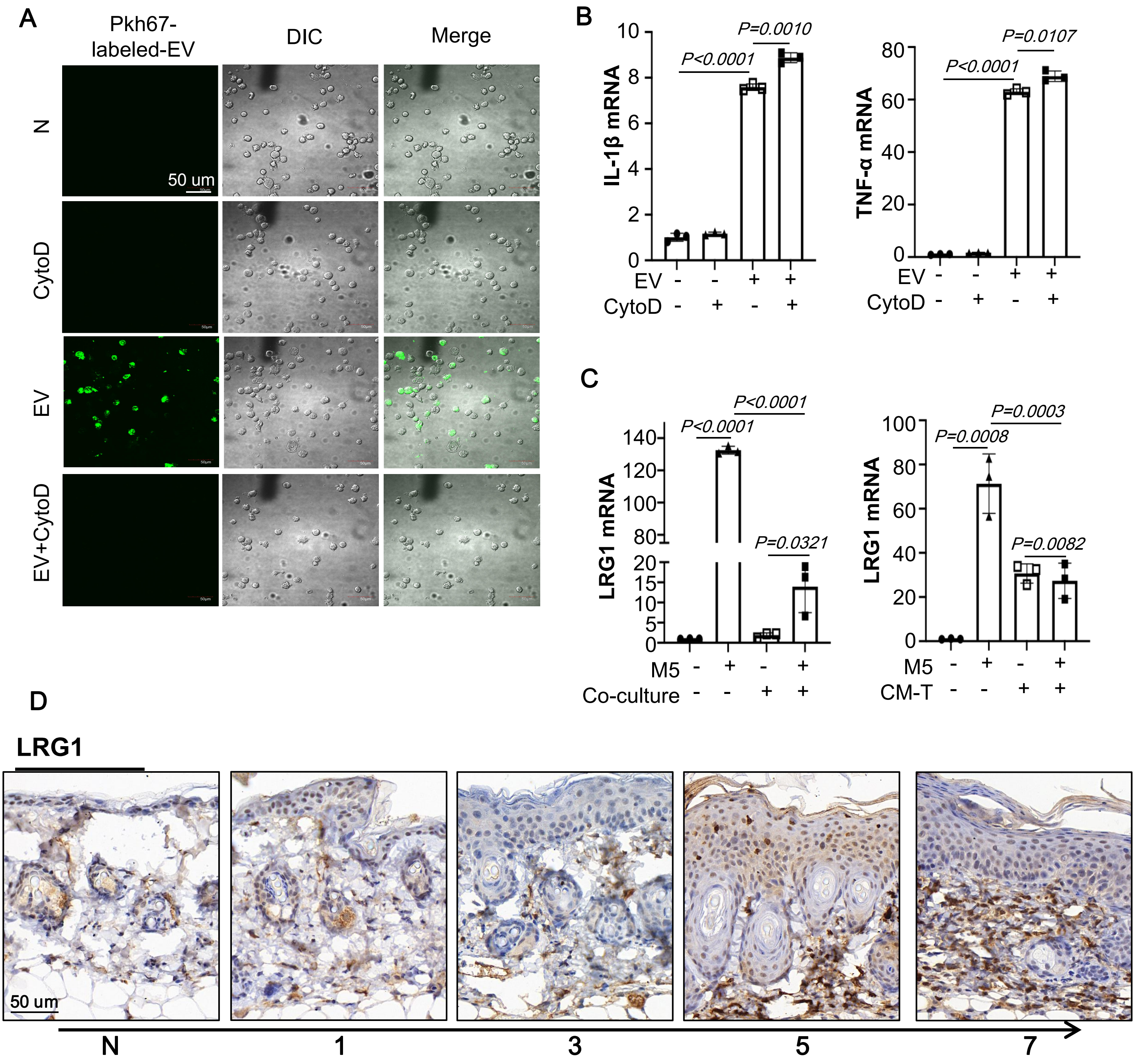

Fig. S3

(A) THP-1 cells were differentiated using PMA treatment for 48 h. Thereafter, THP-1 cells were pretreated with cytochalasin D (1 μM) followed by 18 hour incubation with EV (10^{10}/mL) isolated from M5-treated HaCaT cells. Prior to this incubation, EV were labeled using fluorescent dye PKH67. EV uptake by macrophages was visualized using confocal microscopy.

(B) Differentiated macrophage-like THP-1 cells were pretreated with cytochalasin D (1 μM) for 1 hour followed by incubation with lipotoxic M5-treated HaCaT-derived EV (10^{10}/mL) for 18 hours. Effect of cytochalasin D on TNF-α and IL-1β mRNA levels in THP-1 cells analysed by quantitative realtime PCR.

(C) The level of LRG1 in HaCaT cells of co-culture system and conditioned media culture model analysed by quantitative real-time PCR.

(D) Mice skin tissues stained with LRG1. Scale bar, 50 μm.

Similar results were obtained in 3 independent experiments or in triplicate culture assays.

Supplement Fig. 4. Jiang et al.

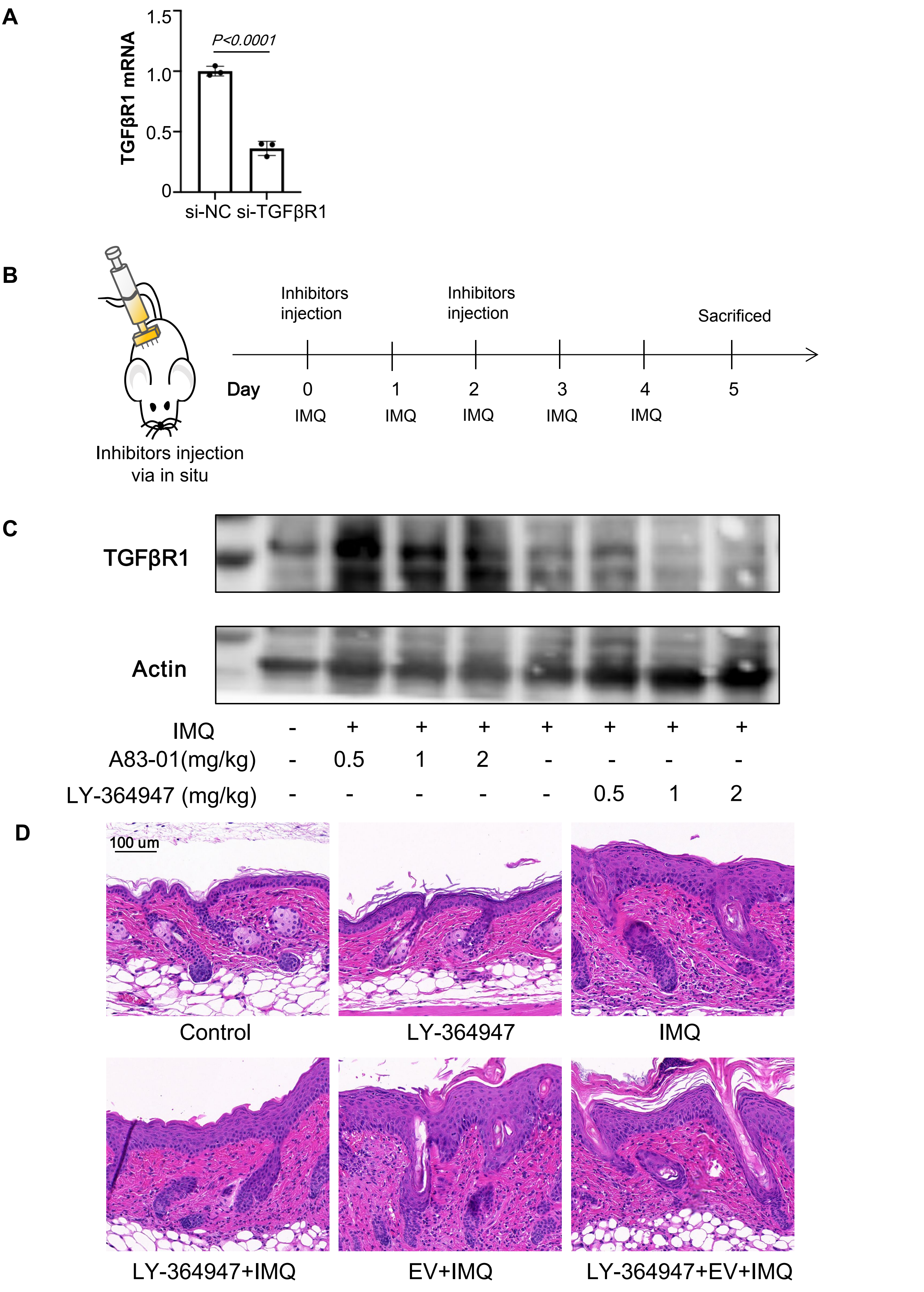

Fig. S4

(A) The level of TGFβR1 in THP-1 cells treated with si-TGFβR1 analysed by quantitative real-time PCR.

(B) Different concentrations of A83-01 and LY-364947 were used to downregulate the expression of TGFβR1 in IMQ-induced mice by skin in situ injection.

(C) Expression of TGFβR1 were detected by western blot.

(E) Mice skin tissues stained with H&E. Scale bar, 100 μm.

Similar results were obtained in 3 independent experiments or in triplicate culture assays.

Table.S1. Primer sequences for quantitative real-time PCR

Supplemental Table 1. Primer sequences

Genes	Forward primer (5'-3')	Reverse primer (5'-3')
**Human**		
β-ACTIN	CATGTACGTTGCTATCCAGGC	CTCCTTAATGTCACGCACGAT
LRG1	GGACACCCTGGTATTGAAAGAAA	TAGCCGTTCTAATTGCAGCGG
TGFβR1	CATTTTTCCCAAGTGCCAGT	ACACCCCTAAGCATGTGGAG
IL-10	GACTTTAAGGGTTACCTGGGTTG	TCACATGCGCCTTGATGTCTG
TNFα	GTACCTCATCTACTCCCAGG	CAGACTCGGCAAAGTCGAGA
IL-1β	CCACAGACCTTCCAGGAGAATG	GTGCAGTTCAGTGATCGTACAGG
IL-23A	CTCAGGGACAACAGTCAGTTC	ACAGGGCTATCAGGGAGCA
MCP1	AGAATCACCAGCAGCAAGTGTCC	TCCTGAACCCACTTCTGCTTGG
IL-8	GAGAGTGATTGAGAGTGGACCAC	CACAACCCTCTGCACCCAGTTT
CD86	CTGCTCATCTATACACGGTTACC	GGAAACGTCGTACAGTTCTGTG
IL-4	CGGCAACTTTGTCCACGGA	TCTGTTACGGTCAACTCGGTG
**Mouse**		
LRG1	TTGGCAGCATCAAGGAAGC	CAGATGGACAGTGTCGGCA
β-actin	GGCTGTATTCCCCTCCATCG	CCAGTTGGTAACAATGCCATGT
IL-23A	AATAATGTGCCCCGTATCCAGT	GCTCCCCTTTGAAGATGTCAG
IL17-A	TCGAGAAGATGCTGGTGGGT	CTCTGTTTAGGCTGCCTGGC
IL-1β	GCAACTGTTCCTGAACTCAACT	ATCTTTTGGGGTCCGTCAACT
IL-10	GCTCTTACTGACTGGCATGAG	CGCAGCTCTAGGAGCATGTG
IL-22	ATGAGTTTTTCCCTTATGGGGAC	GCTGGAAGTTGGACACCTCAA
TGF-B	CTCCCGTGGCTTCTAGTGC	GCCTTAGTTTGGACAGGATCTG
IL-6	TAGTCCTTCCTACCCCAATTTCC	TTGGTCCTTAGCCACTCCTTC

TNF-α	CCTCTCTCTAATCAGCCCTCTG	GAGGACCTGGGAGTAGATGAG
CD86	TGTTTCCGTGGAGACGCAAG	TTGAGCCTTTGTAAATGGGCA
ARG-1	CTCCAAGCCAAAGTCCTTAGAG	AGGAGCTGTCATTAGGGACATC
